# CLINICAL-EPIDEMIOLOGICAL ANALYSIS OF PATIENTS WITH CERVICALGIA AND THE IMPACT OF NUCLEAR RESONANCE

**DOI:** 10.1590/1413-785220253303e287835

**Published:** 2025-08-18

**Authors:** Muryllo Henrique Ferreira de Brito, Karllos Adryano Priscinotte Rodrigues Lima, Pedro Pereira Barbosa, Newton Antônio Tristão

**Affiliations:** 1 Instituto Ortopedico de Goiania (IOG), Goiania, Goias, GO, Brazil.

**Keywords:** Neck Pain, Magnetic Resonance Imaging, Risk Factors, Therapeutic Interventions, Epidemiology, Cervicalgia, Ressonância Magnética, Fatores de Risco, Intervenções Terapêuticas, Epidemiologia

## Abstract

**Objective:**

To analyze the clinical and epidemiological profile of patients with neck pain, assessing risk factors, comorbidities, and the need for therapeutic interventions, as well as the impact of magnetic resonance imaging (MRI) on case management.

**Methods:**

This is a cross-sectional study involving 435 patients treated at the Emergency Department of the Instituto Ortopédico de Goiânia (IOG) between July and December 2023. Sociodemographic, clinical, and risk factor variables were analyzed based on data collected from electronic medical records. Statistical analysis included association tests with a significance level of 5% (p<0.05).

**Results:**

A high prevalence of sedentary lifestyle (94.59%) and prolonged work exposure (78.38%) was observed. Most patients required medication (94.59%), while 63.96% underwent additional clinical interventions. Statistically significant associations were identified between sex, occupational factors, and lesions detected by MRI. The average time to diagnosis was 4.2 months.

**Conclusion:**

Neck pain is strongly associated with modifiable risk factors such as sedentary behavior and occupational overload, highlighting the importance of preventive strategies and individualized therapeutic approaches. MRI played a key role in diagnosis and treatment planning. ***Level of Evidence III; Study of Non-Consecutive Patients; No Uniformly Applied "Gold" Reference Standard***.

## RESUMO

**Objetivo:**

Analisar o perfil clínico-epidemiológico de pacientes com cervicalgia, avaliando fatores de risco, comorbidades e a necessidade de intervenções terapêuticas, além do impacto da ressonância magnética no manejo desses casos.

**Métodos:**

Trata-se de um estudo transversal realizado com 435 pacientes atendidos no Pronto Socorro do Instituto Ortopédico de Goiânia (IOG) entre julho e dezembro de 2023. Foram analisadas variáveis sociodemográficas, clínicas e fatores de risco, com base em dados coletados por meio de prontuários eletrônicos. A análise estatística incluiu testes de associação entre variáveis, com nível de significância de 5% (p<0,05).

**Resultados:**

Observou-se alta prevalência de sedentarismo (94,59%) e exposição prolongada ao trabalho (78,38%). A maioria dos pacientes necessitou de intervenção medicamentosa (94,59%), enquanto 63,96% foram submetidos a intervenções clínicas adicionais. Houve associação estatisticamente significativa entre sexo, fatores ocupacionais e ocorrência de lesões evidenciadas por ressonância magnética. A média de tempo até o diagnóstico foi de 4,2 meses.

**Conclusões:**

A cervicalgia apresenta elevada frequência de fatores de risco modificáveis, como sedentarismo e sobrecarga ocupacional, reforçando a necessidade de estratégias preventivas e abordagens terapêuticas individualizadas. A ressonância magnética contribuiu para o diagnóstico e direcionamento do tratamento. ***Nível de Evidência III; Estudo de Pacientes Não Consecutivos; Sem Padrão de Referência "Ouro" Aplicado Uniformemente***.

## INTRODUCTION

Cervicalgia, characterized by pain in the cervical region of the spine, has emerged as a prevalent health condition in contemporary society. This phenomenon not only affects individuals of various age groups, but also represents a significant challenge for health professionals around the world.^1,2^ In the context of scientific research, understanding the causes, underlying mechanisms and effective strategies for the prevention and treatment of cervicalgia becomes crucial to improve the quality of life of affected individuals and mitigate the socioeconomic impact associated with this condition.^3,4^ In this perspective, this research aims to deepen the clinical, physiopathological and therapeutic aspects related to cervicalgia, providing a solid foundation for future advances in addressing this public health issue.^5,6^

The complexity of cervicalgia transcends the borders of a simple neck pain, demanding a multidisciplinary approach that encompasses areas such as medicine, physiotherapy, psychology, and biomechanical sciences.^7,8^ A deeper understanding of risk factors, including lifestyle, ergonomics, and underlying medical conditions, is essential for a comprehensive and personalized assessment.^9,10^ Furthermore, identifying biomarkers and specific pain patterns associated with cervicalgia can open new perspectives for personalized interventions and targeted therapies, offering a significant potential for improving clinical outcomes and treatment effectiveness.^11^

Cervicalgia is a very prevalent complaint in the general population, and can affect 30 to 50% of the adult population at some point in life.^12^ The main triggers mentioned in the medical literature are repetitive labor, long periods of cervical flexion, increased stress at work, smoking and previous trauma to the neck and shoulders.^13^ In this context, the present research seeks to fill gaps in the current understanding of cervicalgia, promoting the consolidation of scientific knowledge that can guide more effective clinical practices. By analyzing the interconnection between genetic, environmental and behavioral factors, as well as the effectiveness of various therapeutic approaches, this research aims to contribute to a more robust arsenal of preventive and interventionist strategies. The social and clinical relevance of this research transcends the academic scope, reinforcing the need for integrative approaches to better understand and treat cervicalgia in contemporary life.

## METHODOLOGY

### Study design

This study comprises a cross-sectional approach, incorporating quantitative and qualitative elements, with the main objective of analyzing the clinical profile of patients with cervicalgia in Pronto Socorro of the Instituto Ortopédico de Goiânia (IOG).

#### Context

The study covered the period from 1 July 2023 to 31 December 2023, which included 435 patients during the analysis. A consultation of the medical records of the patients treated at emergency room of the IOG was conducted.

#### Participants

All patients over the age of 18 years who underwent MRI with CID of cervicalgia in the period from 1 July to 31 December 2023 were selected.

### Variables

Age, gender, risk factors, in addition to these variables were collected data on personal and family history of neoplasia, time until diagnosis, associated comorbidities such as systemic arterial hypertension, diabetes *melittus*, chronic obstructive pulmonary disease, pulmonary emphysema, tuberculosis, HIV, congestive heart failure, hyperthyroidism, hypothyroidism, atheromatosis, aorta ectasia, osteoporosis, chronic kidney disease, pulmonary thromboembolism.

### Data Source and Measurement

The collection was carried out directly from the hospital system. The evaluation method was through the worksheet in Excel, for qualitative variables were filled 1-yes for present and 2-no for absent, and the numerical variations were adjusted in age in years and time until diagnosis in months. The survey was submitted to *Plataforma Brasil*, with approval in the ethics committee and with CAAE number - 78434824.4.0000.0035.

### Vieses

As for the biases, the bias of selection was minimized as the patients were all from the same hospital and received the same treatment protocol. The bias of information was also minimized because the collection of information was done in the same way for all individuals, as well as the bias of observation which was mitigated because a protocol was developed for collection through a standardized table and a method pre-established by the team. The bias of memory could not be completely minimized because a large part of the records were listed in anamneses, which patients reported some information.

### Study size

The inclusion criteria, which include patients over the age of 18 undergoing resonance imaging as a diagnostic method during the determined period, are clearly outlined. In addition, the exclusion criteria detail specific situations, such as patients under the age of 18, incomplete medical records and cases whose frames are out of the scope of the research, so it was possible to find 435 patients eligible for the study out of a total of 1234 medical records in the sector.

### Quantitative variables

It was necessary to treat the chosen variables, so that the variables were grouped for better development and better analysis.

### Statistical method

#### Statistical method and confusion control

Programs used were Rstudio, Epinfo and Excel. The selection of independent and dependent variables was based on previously established theories and relevant clinical criteria. Statistical tests were performed following the recommended analysis method that was the use of the prevalence ratio with a confidence interval of 95% and a level of significance (p) less than 0.05.

Examination of subgroups and interactions:

It was used test qui square and from the value of *p* selected the statistically significant associations for logistical regression.

#### Missing data

For the missing data, the limitations of the study and the non-referenced were noted.

#### Analytical methods

We chose a stratified random sampling, considering the relevant demographic and clinical characteristics of the target population. This approach sought to ensure equitable representation of important subgroups in statistical analysis.

## RESULTS

In the study period, 1234 magnetic resonance imaging tests were performed. In relation to the age distribution, the patients were aged between 22 and 84, with average and median in 54 years and fashion in 51 years. Although the mean, median and fashion are with similar values, the amplitude and standard deviation indicate that the data present a slight asymmetry to the right. Based on the Shapiro-Wilk test, p = 0.33 is observed, indicating that the data present a distribution tending to normality.

In the comparative analysis of the average age by sex, it is found that the average age of women (53 years) is slightly lower than the average age of men (55 years). However, the t test shows that this difference is not statistically significant (p value = 0.4879). This means that the observed difference may be due to randomness in the sample, and we cannot conclude with confidence that women have a truly different average age than men in the population that these data represent. Despite these findings, the size of the sample is a limitation to safely state that these differences are not present in the profile of patients with similar characteristics.

In relation to work, it was considered patients who remained sitting more than 6 hours a day without getting up, patients with more than 6 hours standing and patients with exhausting hourly loads. Thus, 78.38% of patients had at least one of these exposures in their history and 21.62% did not have this type of exposure.

Sedentarism was considered a factor of high prevalence, with 94.59% of patients versus 5.41% non-sedentary. As diseases and risk factors were considered diabetes, hypertension, obesity, previous injuries, patients with a history of hernia. After the analysis it was possible to verify that 78.38% of patients had any factor involved and 21.62% did not. The average time to diagnosis of injury was 4.2 months with a standard deviation of 6.1 to more or less.

The need for intervention or clinical support were grouped into need for intervention. 63.96% of patients underwent any of these procedures and 36,04% underwent none.

Medical care was present in the clinical history of 94,59% and 5,41% evolved without the need for medications.

By analyzing all the descriptive variables selected related to the outcome, it was possible to identify that of the 258 patients who presented injury, 39% were female and 61% were male. Analyzing the factors related to the work 93% were presented with exposure and 7% did not undergo any exposure, since when we analyzed the associated factors, the majority, 57%, was exposed. In relation to associated treatment, 46% underwent medication and 18% surgery. According to Table 1, the statistical analysis suggests that physical exposure was significantly associated with the presence of injury (Prevalence Ratio = 3.59; 95% CI: 0.92–14.04; p = 0.03), as well as with prognostic risk factors, which showed a strong association with cases that progressed to injury (Prevalence Ratio = 16.43; 95% CI: 2.32–116.53; p = 0.00001). Such findings reinforce the relevance of occupational assessment and early identification of aggravating factors in the management of cervicalgia.

**Table 1:** Assessing the injury and aggravates.

Variable	Injury - yes	Injury - no	Prevalence reason	IC95%	Value p
Physical exposure	92.9%	73.5%	3.59	0.92 - 14.04	0.03*
Associated diseases	92.9%	95.2%	0.74	0.23 - 4.42	0.63
Age	42.9%	48.2%	0.85	0.44 - 1.63	0.62
Adverse prognostic factors	96.4 %	50.6%	16.43	2.32 - 116.53	0.00001*

* Value p <0.05 - statistically significant prevalence ratio.

## DISCUSSION

In 2019, the age-standardized prevalence rate for musculoskeletal disorders, such as neck pain, was 27,0 per 1,000 inhabitants.^14^ Neck pain is one of the most common musculoskeletal disorders and is associated with several risk factors.^11,12^ Among them, psychological factors such as long-term stress, lack of social support, anxiety and depression stand out. In addition, there is evidence suggesting that age and gender may influence the prevalence and development of cervical pain, although more research is needed to confirm these associations.^15^

As for the treatment of acute and chronic musculoskeletal cervical pain, the evidence of pharmacological interventions is limited. In contrast, lumbar pain is the main cause of disability and loss of productivity, and consultation with a specialist in physical medicine and spine rehabilitation within 48 hours for acute pain and within 10 days for all patients with lumbar pain can significantly decrease the rate of surgical interventions.^1,12^

There are few clinical trials dedicated exclusively to the research of cervical pain.^12^ However, non-steroidal muscle relaxants and anti-inflammatory agents have been shown to be effective for acute neck pain. In addition, the exercise is the intervention with the strongest evidence. For conditions such as cervical radiculopathy and facet joint arthropathy, there is limited evidence supporting the use of epidural steroid injections and radiofrequency denervation. Surgery may be more effective in the short term, but not in the long term for most patients. There is substantial heterogeneity between epidemiological studies of cervical pain.^10^ The estimated incidence of cervical pain in 1 year from the available studies ranges between 10.4% and 21.3%, with a higher incidence observed in office and computer workers. The prevalence is generally higher in women, higher in high-income countries and higher in urban areas compared to rural areas.^15^ It is important to note that there is a distinction between neck pain and cervical root pain. Therefore, it is not advisable to deal with both topics in the same book or article.^9^

This study provided a comprehensive and in-depth view of cervicalgia, a prevalent health condition that significantly affects the quality of life of individuals and poses a challenge to health professionals. Through a cross-sectional approach, it was possible to analyze the clinical profile of patients with cervicalgia, considering a variety of variables, including age, gender, risk factors and associated comorbidities.

## CONCLUSION

Cervicalgia is a prevalent and complex health condition, requiring a multidisciplinary approach for its understanding and effective treatment. This research sought to deepen knowledge about the clinical, physiopathological and therapeutic aspects of cervicalgia, highlighting the importance of identifying the risk factors and specific biomarkers associated with this condition. The results revealed a high prevalence rate of factors such as sedentarism and prolonged exposure to work, evidencing the need for preventive and personalized interventions.

However, it is crucial to highlight that the approach of cervicalgia is not limited to pharmacological treatment only, but also includes the promotion of healthy lifestyles, physiotherapeutic interventions and the proper management of related psychological issues. In addition, the research highlights the importance of public education on ergonomics and self-care, aimed at reducing the socioeconomic impact and improving the quality of life of individuals affected by this condition. These findings reinforce the continuing need for integrative studies and approaches to address the challenges associated with cervicalgia and promote more comprehensive and effective cervical health in contemporary life.
